# Nitrogen isotope effects can be used to diagnose N transformations in wastewater anammox systems

**DOI:** 10.1038/s41598-021-87184-0

**Published:** 2021-04-12

**Authors:** Paul M. Magyar, Damian Hausherr, Robert Niederdorfer, Nicolas Stöcklin, Jing Wei, Joachim Mohn, Helmut Bürgmann, Adriano Joss, Moritz F. Lehmann

**Affiliations:** 1 Aquatic and Isotope Biogeochemistry, Department of Environmental Sciences, University of Basel, Basel, 4056 Switzerland; 2 Eawag, Swiss Federal Institute for Aquatic Science and Technology, Dübendorf, 8600 Switzerland; 3 Eawag, Swiss Federal Institute for Aquatic Science and Technology, Kastanienbaum, 6047 Switzerland; 4 Empa, Swiss Federal Institute for Materials Science and Technology, Dübendorf, 8600 Switzerland

**Keywords:** Element cycles, Biogeochemistry, Microbial ecology, Stable isotope analysis, Biogeochemistry, Element cycles, Pollution remediation

## Abstract

Anaerobic ammonium oxidation (anammox) plays an important role in aquatic systems as a sink of bioavailable nitrogen (N), and in engineered processes by removing ammonium from wastewater. The isotope effects anammox imparts in the N isotope signatures (^15^N/^14^N) of ammonium, nitrite, and nitrate can be used to estimate its role in environmental settings, to describe physiological and ecological variations in the anammox process, and possibly to optimize anammox-based wastewater treatment. We measured the stable N-isotope composition of ammonium, nitrite, and nitrate in wastewater cultivations of anammox bacteria. We find that the N isotope enrichment factor ^15^ε for the reduction of nitrite to N_2_ is consistent across all experimental conditions (13.5‰ ± 3.7‰), suggesting it reflects the composition of the anammox bacteria community. Values of ^15^ε for the oxidation of nitrite to nitrate (inverse isotope effect, − 16 to − 43‰) and for the reduction of ammonium to N_2_ (normal isotope effect, 19–32‰) are more variable, and likely controlled by experimental conditions. We argue that the variations in the isotope effects can be tied to the metabolism and physiology of anammox bacteria, and that the broad range of isotope effects observed for anammox introduces complications for analyzing N-isotope mass balances in natural systems.

## Introduction

Anaerobic ammonia oxidation (anammox) has an essential role in the nitrogen (N) cycle as an autotrophic process mediating the conversion of bioavailable forms of N, nitrite (NO_2_^–^) and ammonium (NH_4_^+^), to N_2_, while also regenerating nitrate (NO_3_^–^). It performs this role in both natural and engineered systems. The average contribution of anammox to total N loss in oceanic oxygen minimum zones and marine sediments is likely ~ 30%, controlled by organic C remineralization^1^, and with the balance provided by denitrification. But there are large fluctuations in the denitrification/anammox ratio over space and time. For example, the proportion of anammox has been found to fall between 0 and 79% in marine sediments^2^, and in oceanic oxygen minimum zones its importance also varies tremendously^3^. In engineered settings, the anammox process was first identified in a wastewater treatment system^4^. Today, anammox is commonly used to remove fixed N from high-N ‘sidestream’ wastewater, and the development of a process in which anammox can be used to treat low-N ‘mainstream’ wastewater is the focus of substantial effort^5^. In both natural and engineered settings, it is essential to better understand what environmental factors control the anammox process and its interactions with the rest of the N cycle.

The complete reaction equation for anammox is^6^:

$$1{\text{ NH}}_{4}^{ + } + \, 1.3{\text{ NO}}_{2}^{-} \rightarrow1{\text{ N}}_{2} + \, 0.3{\text{ NO}}_{{3}}^{-}$$

Each of these molecules (NH_4_^+^, NO_2_^–^, NO_3_^–^, N_2_) links anammox to other N cycle processes: (1) denitrification is both a source and a sink for NO_2_^–^, can lead to the generation of NH_4_^+^ through the remineralization of organic matter, and acts as an alternative source of N_2_; (2) aerobic ammonia oxidation is a source of NO_2_^–^ and a competitor for NH_4_^+^; and (3) aerobic nitrite oxidation is a competitor for NO_2_^–^. In natural settings, diffusive gradients of oxygen, organic carbon, and N compounds set up environmental regimes in which anammox bacteria can thrive, while in the context of wastewater treatment, it is necessary to produce such conditions by careful control of process conditions.

To remove fixed N from wastewater, anammox is typically coupled to aerobic ammonia oxidation, which generates nitrite from ammonia, the dominant form of N in wastewater. This paired process is called partial nitritation-anammox (PNA). The complete PNA process requires establishing spatial or temporal gradients in oxygen that enable aerobic ammonia oxidizers to produce nitrite in close proximity to the anaerobic anammox bacteria that metabolize it further. Since anammox is an autotrophic metabolism and only requires partial oxidation of ammonium to nitrite, PNA demands less aeration and consumes less organic carbon than the conventional nitrification–denitrification process for N removal, which includes separate aerobic and anaerobic phases, thereby reducing the overall energy consumption and carbon footprint required for N removal^7^. To date, anammox has proven highly successful in treating sidestream wastewater, with ammonium loads > 500 mg-N/L and at elevated temperatures (> 30 °C)^8^, but it has been difficult to maintain stable anammox operation under the low and variable N loads and ambient temperatures required for treating mainstream wastewater^5^. Stably establishing such balanced gradients, without favoring potential microbial competitors like nitrite-oxidizing or denitrifying bacteria, necessitates the ability to monitor the relative rates of each of these processes, and to understand how each is controlled and how they are interconnected.

Stable isotope measurements are a principal tool for constraining the sources and sinks of different molecules in the N cycle. Specifically, measurement of the variation of ^15^N/^14^N in the molecules NH_4_^+^, NO_2_^–^, and NO_3_^–^ is used to trace and quantify the relative fluxes of the processes that produce and consume these molecules^9^, which can provide insights into both the supply of fixed N to, and its elimination from, an ecosystem. For example, the relative balance of anammox versus denitrification likely sets the N isotope effect of fixed N elimination in the ocean^10^. To accurately make such estimates in complex environmental settings, it is necessary to constrain the stable isotope effects (^15^ε) of individual processes in simpler systems, where individual processes dominate and can be controlled. Isotope effects of N are defined as ^15^ε = (*k*_h_/*k*_l_ – 1) × 1000 (expressed in ‰), where *k*_*h*_ and *k*_*l*_ refers to the reaction rates of the heavy and the light isotopologues, respectively, *e.g.*, ^15^NH_4_^+^ vs. ^14^NH_4_^+^.

Since nitrate is the main reservoir of N in marine and lacustrine settings, using nitrate N and O isotopes to characterize the relative impacts of regeneration, assimilation, and denitrification on its supply is an important tool^11–13^. Ammonium is completely consumed in many ecosystems, but where isotope effects are permitted to be expressed (*e.g.*, anoxic sediments and waters/ocean basins), its δ^15^N is thought to reflect the balance between N fixation, with a ^15^ε near 0‰; aerobic ammonia oxidation, with a variable isotope effect between 14‰ and 46‰^14–17^; and assimilation (4‰ to 27‰)^18^. For anammox, a very limited number of isotope studies have constrained ^15^N/^14^N isotope effects in enriched cultures^10,19^, while one study has estimated their values in wastewater incubations^20^. These studies have established that there are significant variations amongst anammox microbial species with regards to the various N isotope effects associated with specific anammox reactions (*e.g.*, ^15^ε(NH_4_^+^), ^15^ε(NO_2_^–^–N_2_), and ^15^ε(NO_2_^–^–NO_3_^–^)). However, it is not yet well understood how broad this variation can be under environmental conditions, nor what environmental, ecological, or physiological controls are recorded by varying N isotope signatures. In addition, one study has estimated the isotope effects associated with anammox in wastewater.

We have determined the different isotope effects associated with anaerobic ammonium oxidation in mainstream, sidestream, and enrichment cultivations of anammox bacteria derived from a pilot wastewater treatment plant. In the mainstream and enrichment systems, microbial communities were developed as biofilms on biomass-retaining plastic carriers over the course of ≥ 6 months under anoxic, low organic carbon, and relatively high ammonium and nitrite conditions^21^. The resulting microbial community in these mainstream and enrichment systems has been characterized extensively^22,23^. It consists of 10% anammox bacteria by genomic abundance (Fig. S1), but the biovolume of anammox cells exceeds this proportion^22^, and its activity is dominated by anammox^23^. The mainstream and enrichment systems have distinct and stable mixtures of anammox bacteria, including members of the Genera *Candidatus (Ca.)* Brocadia, *Ca.* Kuenenia, and *Ca.* Jettenia^23^. In the sidestream system, anammox-containing granules were enriched under high ambient ammonium concentrations at elevated temperature in a municipal wastewater plant in Thun, Switzerland. The microbial composition of the granules used in this study remains uncharacterized, but a previous study in the same system found that 80% of the granule biomass and 51% of the total biomass consisted of anammox bacteria^24^. Other work on anammox granules suggests that they are also likely to consist of a similar mix of anammox bacteria, heterotrophs and other N cycling bacteria as the anammox-biofilm carrier-bound systems^25^. Since the bacterial community was not restricted to just enriched strains of anammox bacteria, experimental conditions were set in such a way that we were able to look at the anammox dynamics in an isolated manner. These three systems span a range of cell densities, growth rates, and temperature, providing a good opportunity to investigate how some of these conditions can affect the respective N isotope effects.

In each system, we performed several batch fractionation experiments, during which we measured concentrations and stable isotope compositions of nitrite, nitrate, and ammonium during anaerobic ammonium oxidation. Using these measurements, we estimated the ^15^ N/^14^ N isotope effects associated with ammonium and nitrite consumption, and nitrate production. We use these measured isotope effects, coupled to our knowledge of the distinct ensembles of anammox bacteria and growth conditions in each system, to draw conclusions as to the controls on the N isotope effects of anammox; and we discuss the implication of our findings for N isotope dynamics both in engineered systems and in the natural environment.

## Materials and methods

### Mainstream, sidestream, and enrichment anammox cultivations

Mainstream and enrichment experiments were performed with biomass enriched and maintained in sequencing batch reactors on 1 cm × 1 cm × 0.1 cm porous fleece carriers (FLUOPUR, WABAG) for ≥ 6 months under anoxic, organic-matter-poor, nitrite- and ammonium-rich conditions that favor anammox^21^. The microbial community structure of the mainstream system used in these experiments is described in detail in refs. 22 and 23. The carriers contain a mixed microbial community, in which anammox consortia represent 10.4 ± 2.1% (1 s.d.) of the population (Fig. S1), but still dominate the N cycling activity, as confirmed by transcriptome analysis^22,23^. For mainstream experiments, this biomass was maintained in a 8 m^3^ reactor at ambient temperature and dissolved-inorganic-N conditions prescribed by the N load in the municipal waste water (~ 20 mg NH_4_-N/L), while for enrichment experiments the biomass was maintained in a 12 L reactor at elevated nitrite and ammonium concentrations (~ 100 mg NH_4_^+^-N/L) and temperature (~ 34 °C). Sidestream experiments were performed with biomass collected from an active sequencing batch reactor at the wastewater treatment plant Thunersee, Thun, Switzerland. In this material, the anammox biomass has formed concentrated deep-red granules. For sidestream experiment #1, biomass was collected in December 2013 and maintained in an active mode performing PNA (*i.e.*, nitrite and ammonium provided, and anoxic conditions maintained) at the sludge treatment plant in Pfannenstiel, Switzerland, until experiments were performed in April 2014. For sidestream Experiment #2, biomass was collected in May 2019 at the wastewater treatment plant Thunersee, kept at constant temperatures and in active PNA conditions, and used within a week.

### Experimental setup and sampling

All experiments were performed in 12 L bioreactors that were either sealed or purged with a N_2_:CO_2_ or Ar:CO_2_ mixture. During the course of experiments, redox and substrate conditions were controlled to favor anammox over other processes (*e.g.* denitrification). Anoxic conditions were assured by monitoring the redox potential with a redox probe. To ensure that anammox indeed prevailed over denitrification, the reactors were pre-conditioned to minimize labile carbon and nitrate. The temperature settings were 25 °C for mainstream experiments, 30–31 °C for sidestream experiments, and 34–35 °C for enrichment experiments. Experiments were started by adding initial spikes of nitrite and ammonium to reach target concentrations between 25.0 and 195 mg-N/L for ammonium and 17.5 and 113 mg-N/L for nitrite, typically supplying NO_2_^-^ and NH_4_^+^ in the expected anammox stoichiometry of 1.3 nitrite molecules per molecule of ammonium (Table 1). Reactors were then sampled between five and thirteen times, at uniform intervals of ~ 6 to 40 min, depending on the substrate consumption rates observed. Samples were withdrawn from the reactor using a 60-mL syringe, placed into 50 mL Falcon tubes, immediately centrifuged, and the supernatant filtered successively through a 5 µm and a 0.2 µm filter to remove suspended organic matter and bacterial cells. One portion of the sample (1 mL) was treated immediately with 90 µL of 0.6 M sulfamic acid, to remove nitrite; subsequently neutralized with sodium hydroxide; and frozen for later nitrate analysis. The rest of the sample was stored at 4 °C for further sample processing and concentration measurements, as described in the following sections.

**Table 1:** Summary of reaction rates and stoichiometries for all experimental systems. Values are the average of all experiments reported for each system; error estimates are ± 1 standard deviation.

	Ammonium consumption rate, (mg-N L^-1^ min^-1^)	Nitrite consumption rate (mg-N L^-1^ min^-1^)	∆NO_2_^–^/∆NH_4_^+^	∆NO_3_^–^/∆NO_2_^–^	Starting NH_4_^+^ concentrations (mg-N L^-1^)	Starting NO_2_^–^ concentrations (mg-N L^-1^)
Mainstream	0.16 ± 0.02	0.21 ± 0.16	1.30 ± 0.10	− 0.22 ± 0.10	25.0 to 56.0	17.5 to 43.6
Enrichment	0.79 ± 0.16	1.16 ± 0.79	1.42 ± 0.16	− 0.22 ± 0.05	63.8 to 195	45.9 to 113
Sidestream	0.49 ± 0.07	0.68 ± 0.49	1.37 ± 0.09	− 0.162 ± 0.002	61.4 to 87.1	72.8 to 85.0

### Concentration measurements

Ammonium and nitrite concentrations were measured immediately after sampling using colorimetric chemical techniques. Nitrate concentrations were measured on the sulfamic acid-treated subsample by reaction with V(III) at 95 °C and detection by chemiluminescence^26^.

### Isotopic measurements

For the measurement of nitrite N isotopes, nitrite was converted to N_2_O by reaction with sodium azide^27,28^. For this, immediately after sampling, enough sample to yield 80 nmol of N_2_O was place into nitrite-free seawater in a 20 mL glass vial, which was then sealed with a gas-tight rubber septum. Then, 0.3 mL of a 1:1 mixture of 2 M sodium azide (Sigma-Aldrich, ≥ 99.0% pure) and 20% v/v acetic acid (Sigma-Aldrich, ReagentPlus grade), previously degassed for > 1 h with N_2_, was added using a syringe and needle and the sample was shaken well. After > 30 min, the reaction was stopped by addition of 0.2 mL 10 M NaOH (Sigma-Aldrich, ACS Reagent grade) and the sample vial was stored cap-down until analysis. Measurements were corrected and standardized relative to N_2_ in air using blanks and international nitrite standards (N7373, δ^15^N_AIR_ = − 79.6‰, and N10219, δ^15^N_AIR_ = 2.8‰)^29^ prepared at the same time and in the same way as the samples.

For the measurement of ammonium N isotopes, ammonium was converted to N_2_O by reaction with hypobromite to form nitrite, which was subsequently converted to N_2_O using the sodium azide method^30^. Samples were stored at 4 °C until ready for preparation. Before hypobromite treatment, a defined volume of sample (40 nmol ammonium) was topped up to 5 mL with Milli-Q water, equivalent to 8 µM ammonium. Then, to remove pre-existing nitrite, all samples were treated with an amount of 1 mM sulfamic acid equal to 1.5 × the measured amount of nitrite in that sample, plus 46 µL of 6 M HCl to yield a pH of 2–3, and allowed to react overnight at room temperature. To each sample, 0.5 mL of an alkaline BrO^-^ solution, prepared immediately before use as described in ref. 30, was added. After 30 min, the reaction was stopped by addition of 0.1 mL of 0.39 M sodium arsenite. The pH of the solution was brought to a value between 5 and 11 by addition of ~ 0.36 mL 6 M HCl, and then the sample was treated according to the azide method to convert nitrite to N_2_O, as described above. Measurements were corrected and standardized using blanks and the international ammonium standards IAEA-N1 (δ^15^N_AIR_ = 0.4‰), IAEA-N2 (δ^15^N_AIR_ = 20.3‰), and USGS26 (δ^15^N_AIR_ = 53.7‰), prepared at the same time and in the same way as the experimental samples.

Nitrate N isotopes were measured using the bacterial denitrifier technique^31–33^. Briefly, enough nitrate sample to yield 50 nmol of N_2_O was injected into concentrated and helium-purged aliquots of *Pseudomonas chlororaphis* subsp. *aureofaciens* (ATCC#13,985), which converts all nitrate that is supplied to N_2_O. Measurements were corrected using blanks and the international nitrate standards IAEA-N3 (δ^15^N_AIR_ = 4.7‰) and USGS34 (δ^15^N_AIR_ = − 1.8‰), and our internal laboratory standard UBN-1 (δ^15^N_AIR_ = 14.15‰).

Nitrous oxide samples from the azide, hypobromite, and denitrifier method were all measured by continuous-flow gas source isotope ratio mass spectrometry coupled to a custom cryogenic purge-and-trap system^34^. All N isotope measurements are reported on the AIR-N_2_ scale, where R_i_ is ^15^ N/^14^ N, in per mil (‰), according to the following definition:

$$\delta^{15} N = \left( {\frac{{R_{{{\text{sample}}}} }}{{R_{{{\text{air}}}} }} - 1} \right) \times 1000.$$

## Results

### Substrate concentrations, reaction rates, and stoichiometry

**Figure 1:**
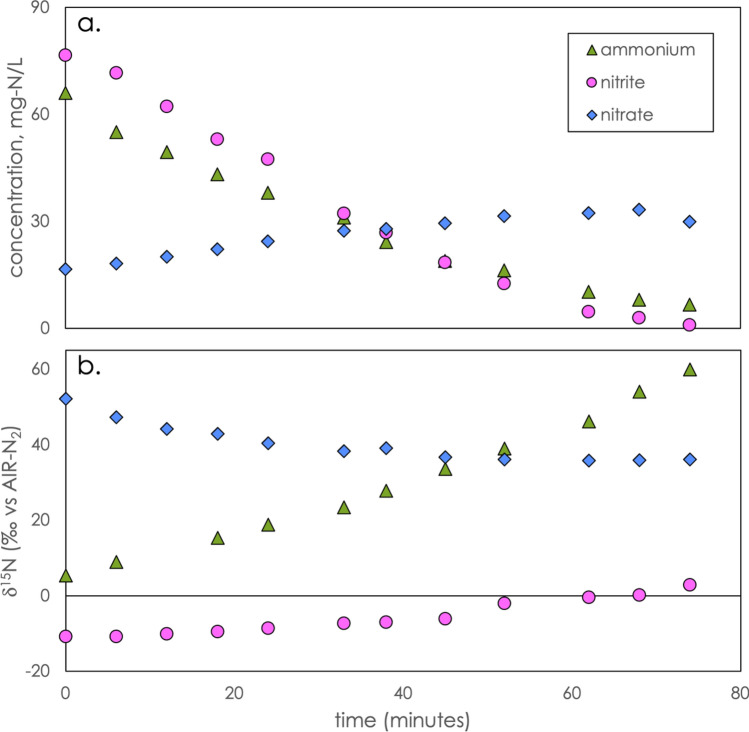
The variation in concentration (**a**) and N isotopic composition (**b**) of substrates (NH_4_^+^ and NO_2_^–^) and product (NO_3_^–^) over the course of a typical anammox incubation experiment (exemplary experiment with enrichment culture, 28 January 2019). Note that the nitrate δ^15^N is not corrected for the fraction of nitrate that was already present at the beginning of the incubation (see text).

Figure 1a displays the typical temporal trend for NH_4_^+^, NO_2_^-^, and NO_3_^-^ concentrations after spiking anammox incubations with NH_4_^+^ and NO_2_^-^. As expected for anammox, we observed decreasing concentrations of ammonium and nitrite accompanied by an increase in nitrate. From the concentration change over time, we calculated batch reaction rates for each experiment, which are summarized in Table 1. We found that the highest substrate consumption rates were in the enrichment system, which is operated at the highest temperatures and starting concentrations, and has been enriched with a focus on maximizing ammonium and nitrite consumption rates. The sidestream system, which is maintained at intermediate temperatures and initial nitrite and ammonium concentrations, and the mainstream system, at the lowest temperatures and substrate concentrations, were observed to yield successively slower rates.

The observed decrease in nitrite and ammonium on the one hand, and increase in nitrate on the other, gives the stoichiometry of the anaerobic oxidization of ammonium in a particular experiment. We found ∆[NH_4_^+^]/∆[NO_2_^–^] ratios of 1.42 ± 0.16 for enrichment experiments, 1.30 ± 0.10 for mainstream experiments, and 1.37 ± 0.09 for sidestream experiments (Table 1). The ∆[NO_3_^–^]/∆[NO_2_^–^] ratio was − 0.22 ± 0.05 for enrichment experiments, − 0.22 ± 0.10 for mainstream experiments, and − 0.162 ± 0.002 for sidestream experiments. Thus, with some degree of variability, the anaerobic oxidation of ammonium with nitrite followed the previously observed stoichiometry of anammox, confirming that anammox is the dominant N transforming pathway in our incubations under the provided redox and substrate conditions^35,36^.

### Estimating the N isotope effects in ammonium

The N isotope trends for an example anammox experiment are depicted in Fig. 1b. As ammonium is consumed by anammox, δ^15^N of the remaining substrate increases, indicating the preferential oxidation of ^14^NH_4_^+^ to N_2_, which is represented by a positive value of the isotope effect ^15^ε(NH_4_^+^). The value of ^15^ε(NH_4_^+^) was calculated according to a Rayleigh fractionation model, which assumes that ammonium is continually removed from a uniform reservoir. The model is implemented by plotting the stable isotope composition, δ^15^N(NH_4_^+^), against the natural logarithm of the fraction of remaining substrate at a given time, relative to the NH_4_^+^ concentration at the beginning of the experiment, [NH_4_^+^]/[NH_4_^+^]_0_. Then, the isotope effect *ε* was determined using a linear regression, according to the equation:

$$\delta^{15} N_{i} = \varepsilon^{15} \left( {{\text{NH}}_{4}^{ + } } \right) \cdot \ln \frac{{\left[ {{\text{NH}}_{4}^{ + } } \right]}}{{\left[ {{\text{NH}}_{4}^{ + } } \right]}}_{0} + \delta^{15} N_{0},$$

where δ^15^N_0_ refers to the N isotopic composition of the ammonium at the beginning of the experiment, and δ^15^N_*i*_ reflects the N isotopic composition of the substrate at any given time point^14^. The uncertainty in ^15^ε(NH_4_^+^) for an individual experiment is given by the standard error of the slope in the linear regression model, and ranged from 0.4 to 10.3‰, but was typically below 1.5‰ (Fig. S2, Supplementary Datafile).

**Figure 2:**
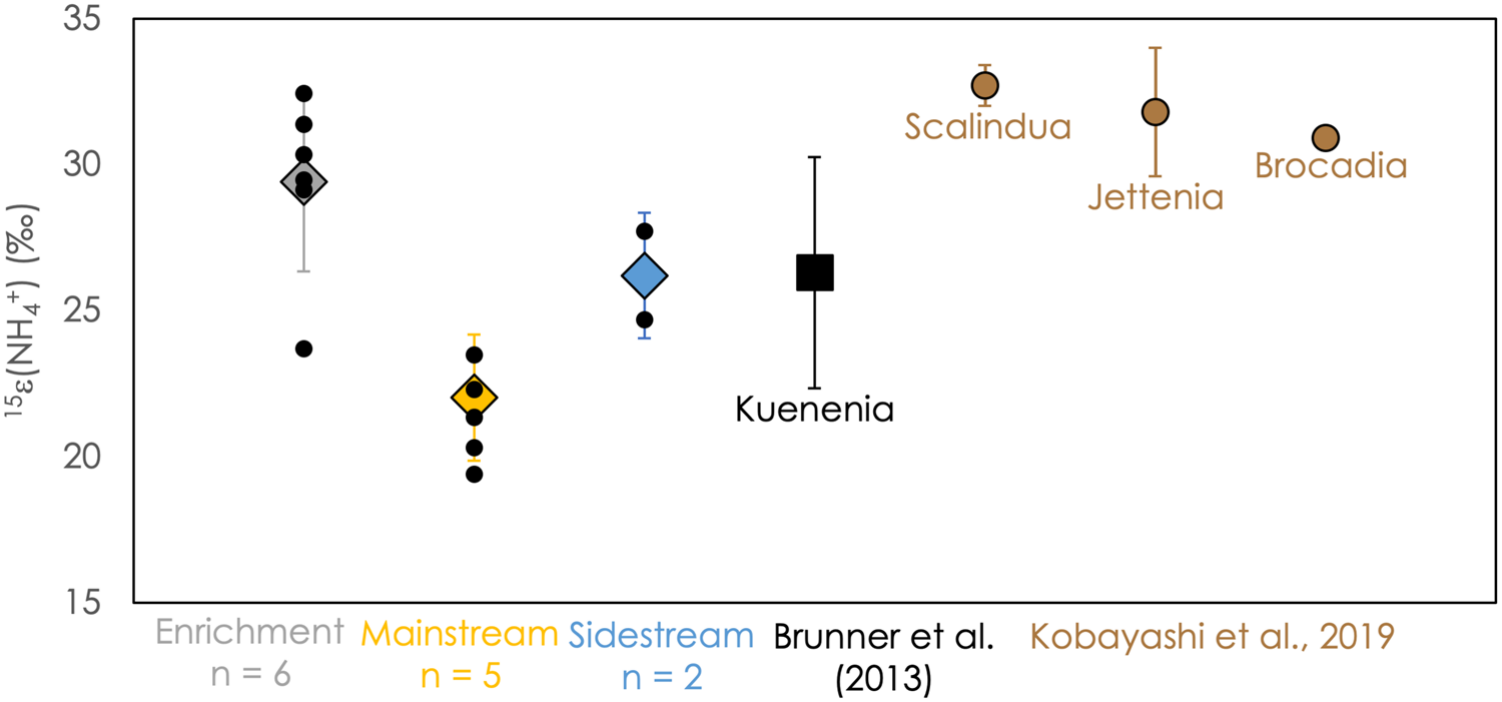
The N isotope effect associated with the oxidation of ammonium, ^15^ε(NH_4_^+^), for all experiments. Colored diamonds represent average values for different anammox incubations measured in this study, with 1-standard-deviation error bars. Small black circles represent the results of individual experiments. For comparison, the results of Brunner and coworkers (black square) and Kobayashi and coworkers (brown circles) are also shown^10,19^.

Figure S2 shows the Rayleigh distillation plots for all experiments reported here. The isotope effect for ammonium consumption,^15^ε(NH_4_^+^), ranged between 19.4 and 32.4‰, as shown in Fig. 2. ^15^ε(NH_4_^+^) values for particular anammox cultivations exhibit a significantly narrower range. For example, experiments performed under mainstream conditions revealed a ^15^ε(NH_4_^+^) of 22.0 ± 2.1 ‰, while experiments performed under enrichment conditions displayed an N-isotope effect of 29.4 ± 3.4 ‰ (± 1 s.d.).

### Estimating the N isotope effects in nitrate and nitrite

**Figure 3:**
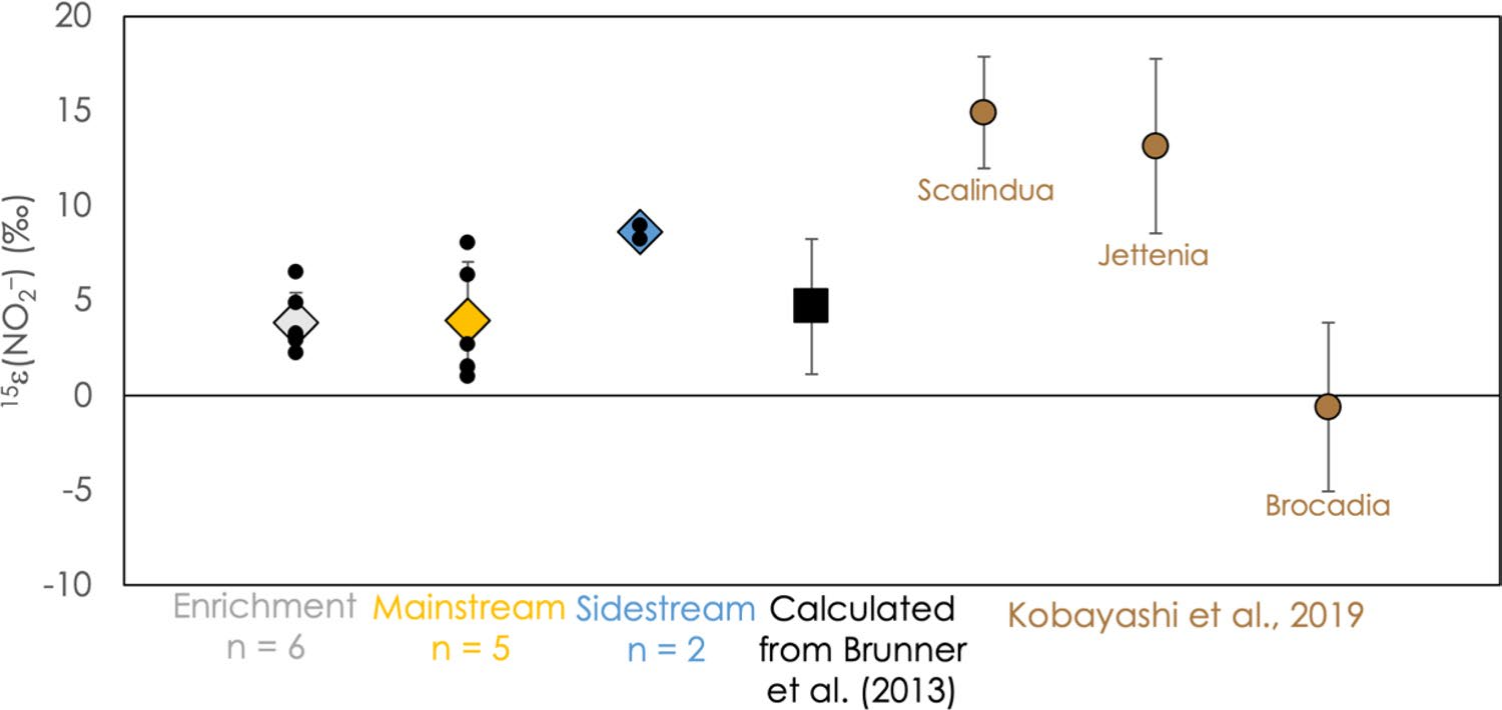
The N isotope effect associated with the consumption of nitrite, ^15^ε(NO_2_^–^), reflecting the composite effect of conversion both to nitrate and N_2_, for all experiments. Colored diamonds represent average values, surrounded by 1-standard-deviation error bars. Small black circles represent the results of individual experiments. For comparison, the results of Brunner and coworkers^10^ (black square) and Kobayashi and coworkers^19^ (brown circles) are also shown.

Figure 1b displays typical nitrite and nitrate N isotopic trends over the course of an experiment. Determining isotope enrichment factors associated with the consumption of nitrite and the production of nitrate requires considering both processes together. Nitrite is consumed by both reductive and oxidative pathways, so the simplifying assumptions of the Rayleigh model (*i.e.*, assuming a single irreversible reaction) may not be valid. Nevertheless, the model adequately describes nitrite consumption in our experiments, and we therefore calculated ^15^ε(NO_2_^–^) as described in the preceding section for ammonium (Fig. S3). Values of ^15^ε(NO_2_^–^) ranged from 1‰ to 8‰, as shown in Fig. 3. This parameter reflects the imprint of anammox on the external pool of nitrite, which can be regarded as a composite isotope effect that convolves nitrite reduction to N_2_ and nitrite oxidization to nitrate, as well as the ratio between these two processes.

**Figure 4:**
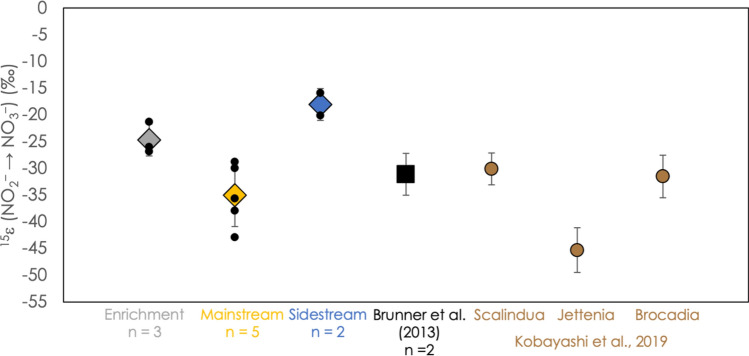
The N isotope effect associated with the oxidation of nitrite to nitrate, ^15^ε(NO_2_^–^–NO_3_^–^), for all experiments. Colored diamonds represent average values, surrounded by 1-standard-deviation error bars. Small black circles represent the results of individual experiments. For comparison, the results of Brunner and coworkers^10^ (black square) and Kobayashi and coworkers^19^. (brown circles) are also shown.

To estimate the isolated isotope effect associated with nitrite oxidation to nitrate, we used measurements of the δ^15^N of nitrate and followed the approach described by Brunner and coworkers^10^. First, to account only for nitrate generated in the course of a particular experiment, nitrate δ^15^N values were corrected for the amount and isotopic composition of nitrate present at the start of the experiment. After correction, δ^15^N(NO_3_^–^) values, expressed relative to the δ^15^N of the nitrite present at the start of the experiment, were plotted against the parameter F (Fig. S4), which quantifies the accumulation of a product in a Rayleigh distillation process and is given as^10^:

$$F = 1 + \frac{f \cdot \ln f}{{1 - f}},$$

where *f* is the proportion of nitrite remaining, [NO_2_^–^]/[NO_2_^–^]_0_. In this space the negative of the y-intercept corresponds to the isotope effect for the formation of nitrate from nitrite, ^15^ε(NO_2_^–^–NO_3_^–^), and so is determined for each experiment, along with its associated uncertainty (one standard error confidence internal), using a linear regression (summarized in Fig. 4). We find that ^15^ε(NO_2_^–^–NO_3_^–^) varies between − 15.9‰ and − 44.9‰ in the experiments described here, but each given experimental setting displays a much narrower range, with values of – 35.0‰ ± 5.8‰ for mainstream experiments, − 24.7‰ ± 3.0‰ for enrichment experiments, and − 18.0‰ ± 3.0‰ for sidestream experiments (± 1 s.d.). Also indicated in Fig. 4 are values for ^15^ε(NO_2_^–^–NO_3_^–^) based on previous enrichment culture measurements for various anammox bacteria^10,19^.

Then, the two measured parameters, ^15^ε(NO_2_^–^) and ^15^ε(NO_2_^–^–NO_3_^–^), and the branching ratio *x,* the proportion of nitrite oxidized to nitrate in a given experiment, were used to calculate ^15^ε(NO_2_^–^–N_2_), after rearranging the following equation:

$$^{15} \varepsilon \left( {{\text{NO}}_{2}^{-} } \right) = x \cdot^{15} \varepsilon \left( {{\text{NO}}_{2}^{-} {-}{\text{NO}}_{3}^{-} } \right) + \left( {1 - x} \right) \cdot^{15} \varepsilon \left( {{\text{NO}}_{2}^{-} {-}{\text{N}}_{2}^{{}} } \right).$$

**Figure 5:**
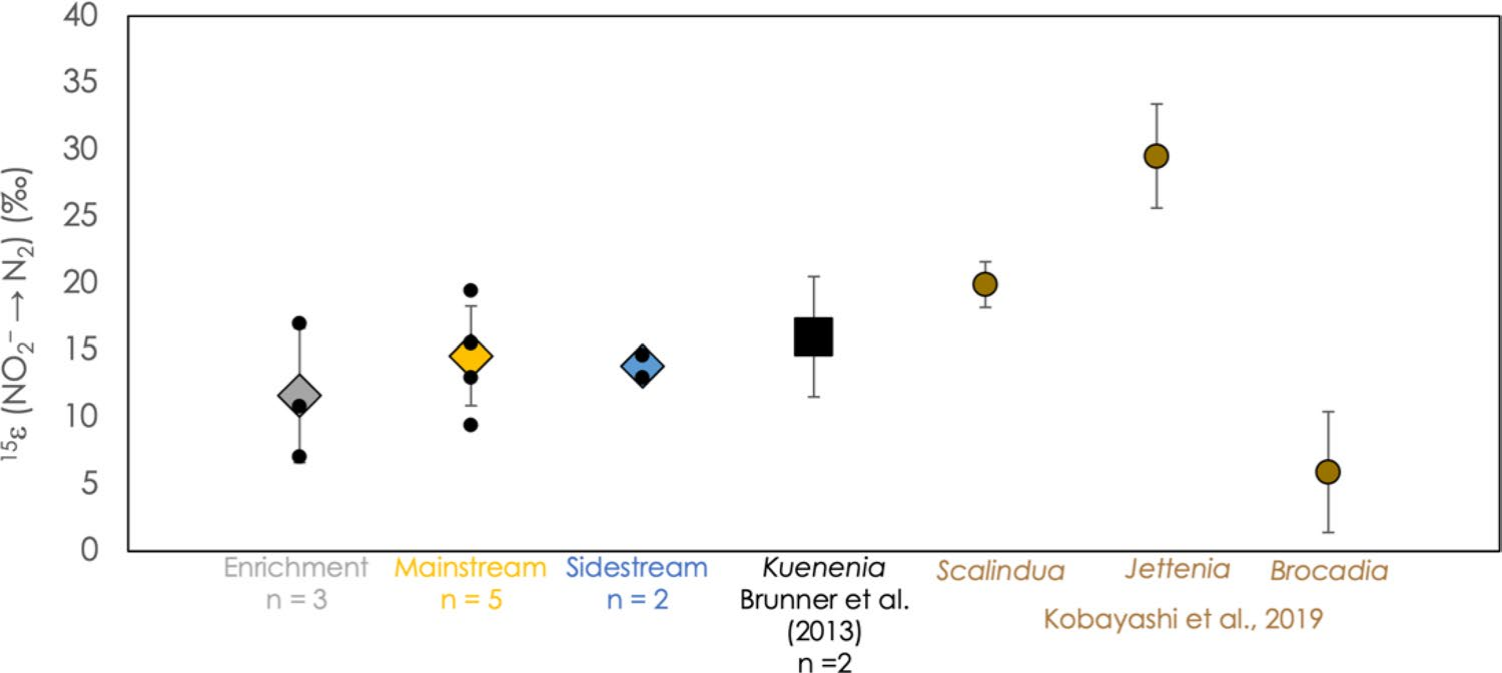
The N isotope effect associated with the reduction of nitrite to N_2_, ^15^ε(NO_2_^–^–N_2_), for all experiments. Colored diamonds represent average values, surrounded by 1-standard-deviation error bars. Small black circles represent the results of individual experiments. For comparison, the results of Brunner and coworkers^10^ (black square) and Kobayashi and coworkers^19^ (brown circles) are also shown.

The parameter ^15^ε(NO_2_^–^–N_2_) was consistent across all conditions, with an average value of 13.5‰ ± 3.7‰ for all experiments, 14.6‰ ± 3.7‰ for mainstream experiments, 11.6‰ ± 5.0‰ for enrichment experiments, and 13.8‰ ± 1.2‰ for sidestream experiments (± 1 s.d.; Fig. 5).

## Discussion

### Variation in the N isotope effect imparted by ammonium oxidation

**Figure 6:**
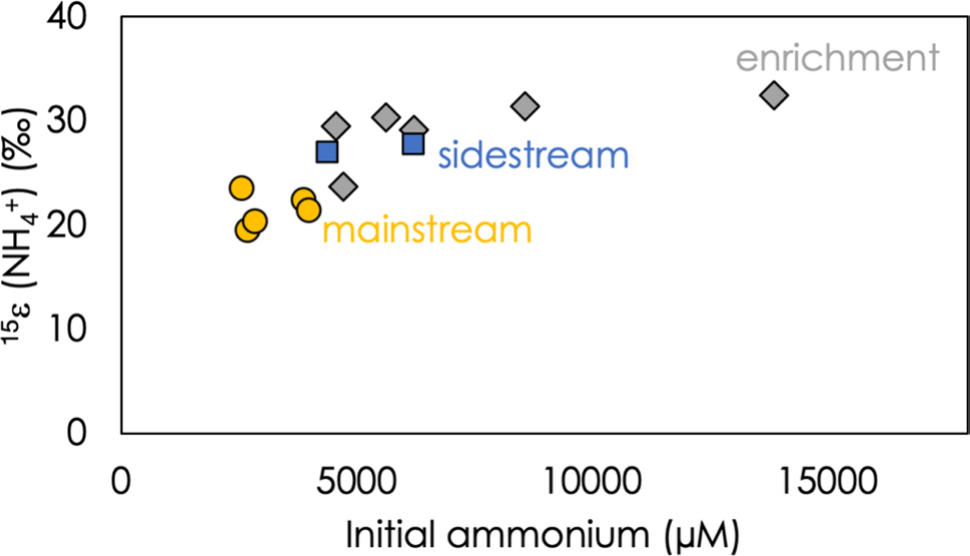
^15^ε(NH_4_^+^) for each individual experiment, compared to the concentration of ammonium at the start of that experiment. Mainstream experiments are plotted in yellow circles, sidestream experiments in blue squares, and enrichment experiments in grey diamonds.

While the ammonium isotope effect, ^15^ε(NH_4_^+^), varies by over 13‰ across all experiments, it exhibits a narrower range for a specific experimental setting with distinct cultivation conditions (mainstream, enrichment, sidestream), it exhibits a broader range across all experiments (Fig. 2). There are a number of possible physiological and experimental conditions that differ among experiments, including reaction rate, temperature, and initial concentration of substrates. As shown in Fig. 6, there is a systematic decrease in ^15^ε(NH_4_^+^) at decreasing initial ammonium concentration, while at the highest ammonium concentrations, the value of ^15^ε(NH_4_^+^) appears to plateau at a value near 32‰, close to the maximum value (32.7 ± 0.7‰) observed by Kobayashi and coworkers in chemostat experiments with enriched cultures^19^. Typically, the isotope effect imparted into a substrate pool by a kinetic process is set at the first irreversible step; any isotope effects that occur before this point can be expressed, while any that occur after it are concealed^37,38^. Substrate supply limitations can decrease the reversibility of a given step, and thereby let it modulate the net isotope effect of a multi-step process. This behavior has been proposed to play important roles in controlling the isotopic signatures of microbial sulfate^39,40^ and nitrate^41,42^ reduction.

In the case of anammox bacteria, this pattern suggests that the relative kinetics of ammonium uptake and oxidation control the observed value of ^15^ε(NH_4_^+^). The typical path of an ammonium molecule through an anammox cell requires crossing several cell membranes to the eventual site of reaction, inside the anammoxosome^43,44^. When ammonium concentrations are relatively high and ammonium oxidation is not uptake-limited, ^15^ε(NH_4_^+^) is set at the hydrazine synthase (Hzs) enzyme, at which ammonium binds and is subsequently oxidized to hydrazine^43^. The maximum observed value of ^15^ε(NH_4_^+^) would then be the expression of all isotope effects up to, and including, this bond-breaking step. But if, at relatively low ammonium concentrations in the external medium, the rate of ammonium oxidation is limited by its uptake, then active transport or passive diffusion of ammonium will become the first irreversible step and thereby set the observed value of ^15^ε(NH_4_^+^). Indeed, for assimilatory uptake by a marine bacterium, ^15^ε(NH_4_^+^) has been shown to depend on the external ammonium concentration, varying from 3.8 to 26.5‰ across an ammonium concentration range of 0.3 to 316 mg-N/L, as the first irreversible step changes from active transport at low NH_4_^+^ concentrations to diffusion at intermediate concentrations and the enzymatic reaction associated with assimilation at high concentrations^18^.

In the process of NH_4_^+^ uptake by anammox bacteria, a number of steps could imaginably impact the isotope effect imparted on the ammonium pool. Prior to oxidation within the anammoxosome, ammonium must cross three membranes to reach the Hzs enzyme^44^. This is distinct from many other respiratory processes in the N cycle, including aerobic ammonia oxidation, where ammonia is thought to be oxidized in the periplasm^45,46^. Metagenomic characterization of anammox bacteria in the mainstream system used in these experiments reveals the presence of genes for *amt* ammonium transporters in these species^23^, while past studies of *Ca.* Kuenenia stuttgartiensis shows that anammox bacteria feature a number of genes homologous to those that express AmtB ammonium transporters in other bacteria^47,48^. These transporters could function to transport ammonium across two membranes, first into the cytoplasm and then into the anammoxosome^49^. In addition, passive, diffusive influx of ammonium into the cell could play a role, especially at relatively high external ammonium concentrations. Therefore, it is easily imaginable that under different physiological conditions, the observed value of ^15^ε(NH_4_^+^) could reflect (1) free motion of ammonium into the anammoxosome and the full expression of the isotope effect associated with NH_4_^+^ oxidation, (2) irreversibility in either of two active transport steps, or (3) irreversibility of diffusive transport of ammonium into the periplasm.

We do not know the N isotope effects for ammonium diffusion into the cell and ammonium transport to the anammoxosome. But in analogy to considerations made for the diffusion and active transport of nitrate and active transport into the cells of denitrifying bacteria^41^, we conclude that it is reasonable to expect the N isotope effects for both passive ammonium diffusion and active transport to be much smaller that the enzyme-level N isotope effect associated with the actual ammonium oxidation. Therefore, when external ammonium is relatively low, and NH_4_^+^ transport becomes the rate-limiting step in anaerobic ammonium oxidation, the overall N isotope effect will approach that associated with NH_4_^+^ uptake or transport, and will likely be lower than under NH_4_^+^-replete conditions, where the full expression of the isotope effect associated with NH_4_^+^ oxidation may be expressed. This also supports the observation (Fig. 6) of decreasing ^15^ε(NH_4_^+^) under decreasing ammonium availability.

Importantly, in a different microbial setting, *e.g.,* in an oceanic environment, which has anammox bacteria with different (*e.g.*, higher) affinities for ammonium uptake and oxidation, we predict that the same endmember values of ^15^ε(NH_4_^+^) that are seen in these experiments will be observed, but with the relationship between them unfolding at different (*e.g.,* lower) values of ammonium concentration, cell densities, and in turn different cell-specific anammox rates. It is also important to note that the cell-specific anammox rate, not the bulk reaction rate, is the essential parameter for understanding the balance between the different processes at work. Unfortunately, because of the biofilm-dwelling nature of the anammox communities in this study, it is challenging to estimate accurately the number of anammox cells present, and so we were not able to determine the cell-specific anammox rate in our experimental setup. Yet, even at substrate concentrations that are much higher than those typically found in the natural environment, NH_4_^+^ uptake can be limiting if the bacterial cell density is high, as is the case in this study.

For understanding the role that the balance between ammonium uptake and oxidation may play in controlling ^15^ε(NH_4_^+^), it is useful to compare anammox bacteria to aerobic ammonia oxidizing bacteria (AOB) and archaea (AOA). It is notable that the range of ^15^ε(NH_4_^+^) is similar for anammox bacteria and aerobic ammonia oxidizers; the AOB and AOA express values of ^15^ε(NH_4_^+^) between 14 and 42‰^14–17^. AOB and AOA perform catabolic ammonia oxidation using the ammonia monooxygenase enzyme. In AOB, this enzyme is located in the periplasm^45,46^, not in an internal cell structure like the anammoxosome, and so it is unlikely that active transport controls observed isotope effects. Instead, it has been proposed that variations in ^15^ε(NH_4_^+^) for the AOB are related to sequence variations in ammonia monooxygenase^16^. But recent results from Kobayashi and coworkers suggest that ^15^ε(NH_4_^+^) for anammox bacteria is species-independent; for the three different species tested under similar experimental conditions, the N isotope effects were consistent^19^. In our experiments, variations in the ^15^ε(NH_4_^+^) values were observed in mainstream and enrichment experiments, where the anammox bacteria population is expected to be similar, which also argues against species dependence. Indeed, the Hzs enzyme seems well conserved across anammox clades^50^, and, therefore, the ammonium N isotope effect variation observed here cannot be attributed to sequence variations, and is more likely due to the changing experimental conditions (NH_4_^+^ concentrations), as discussed above.

Irrespective of the explanations for the observed N-isotope effect variability for both ammonium oxidation modes, the overlap in the ranges of values for ^15^ε(NH_4_^+^) for anammox and aerobic ammonia oxidation suggests that in a system that might be either aerobic or anaerobic, the mechanism of ammonium oxidation cannot necessarily be identified based on the ammonium N isotope signature. That is, an enrichment in ^15^ N associated with ammonium consumption cannot be attributed to ammonia oxidizing bacteria or anammox bacteria based on this measurement alone. Further work to compare the responses of both aerobic ammonia oxidizers and anammox bacteria to changing concentrations and cell-specific reaction rates would be helpful for identifying the overall environmental controls on ^15^ε(NH_4_^+^) under both oxic and anoxic conditions.

### ^15^ε(NO_2_^–^) reflects a mixture of processes

The parameter ^15^ε(NO_2_^–^) reflects the weighted sum of the isotope effects for the consumption of nitrite by reduction to N_2_ and by oxidation to nitrate, and so its value depends on these two processes, as well as upon the stoichiometric ratio between them. For considering the physiology of anammox and its role in a biogeochemical N cycling network, it is of limited use, but in a system where the δ^15^N of nitrite can be readily measured it is valuable to know how to interpret it. It exhibits relatively little variation across the experimental conditions described here, and is also consistent with the result reported by Brunner and coworkers for *Ca.* K. stuttgartiensis enrichment cultures (Fig. 3)^10^, but our results differ from ^15^ε(NO_2_^–^) values for other anammox species by Kobayashi and coworkers^19^. The principal cause of the constancy of ^15^ε(NO_2_^–^) in these experiments is likely the stability of ^15^ε(NO_2_^–^–N_2_) across all experiments, and is discussed in the sections that follow.

### The N isotope effect associated with the reduction of nitrite by anammox, ^15^ε(NO_2_^–^–N_2_), reflects the microbial community

The N isotope effect associated with the reduction of nitrite to N_2_ in anammox, ^15^ε(NO_2_^–^–N_2_), is consistent across all three experimental settings (Fig. 5), which is notable when compared to the broad range in ^15^ε(NO_2_^–^–N_2_) observed in previous pure culture experiments with members of the genera *Ca.* Kuenenia*, Ca.* Scalindua*, Ca.* Jettenia*,* and *Ca.* Brocadia^10,19^, as well as in anammox incubation experiments^20^. This consistancy is also striking in light of the variation of ^15^ε(NH_4_^+^) observed in this study, and suggests that variations in substrate concentrations, reaction rates, or other physiological conditions are not strong controls on ^15^ε(NO_2_^–^–N_2_). Instead, the identity of the anammox bacteria, and in turn its biochemical processing of nitrite, appears to exert control over this isotope effect. In the mainstream system used for these experiments, it has been shown that species in the genus *Ca.* Brocadia are the principal members of the anammox community present, but that *Ca.* Kuenenia and *Ca.* Jettenia are also represented (Table S1)^23^. Indeed, using the metagenomic characterization of the mainstream system reported by Niederdorfer and coworkers^23^, as well as the observed values of ^15^ε(NO_2_^–^–N_2_) from previous studies^10,19^, we calculate an expected value of ^15^ε(NO_2_^–^–N_2_) for the mainstream system of 7.5‰ ± 5.5‰ (1 s.d.). This result is close to, but distinct from, the observed value, and leads to the conclusion that ^15^ε(NO_2_^–^–N_2_) in these systems is the result of a stable mixture of different anammox species, but that the contribution of different species to anammox activity under a specific set of experimental conditions may not directly reflect their cellular abundance. Likewise, although we do not yet know the microbial community composition of the material used in the sidestream experiment, we predict, based on its consistent value for ^15^ε(NO_2_^–^–N_2_), that it is similar to that seen in the mainstream and enrichment settings, and we speculate that this microbial community has been stable over the course of the ~ 5 years between 2014 and 2019.

The distinct values of ^15^ε(NO_2_^–^–N_2_) observed for different species can be connected to key variations in the anammox metabolism. Although the canonical anammox mechanism includes the reduction of nitrite to NO by a nitrite reductase enzyme^51^, genomes of anammox bacteria of the Genus *Ca.* Brocadia^52,53^, including 5 of 6 metagenome-assembled genomes for bacteria in the mainstream system used in this study^23^, typically lack any canonical nitrite reductase in their genomes. Instead, it has been proposed that *Ca.* Brocadia do not produce NO and instead have hydroxylamine as the intermediate between nitrite and hydrazine^52^. This hypothesis is supported further by the nature of Hzs, which has two catalytic centers, one of which reduces NO to hydroxylamine, while the second conproportionates hydroxylamine and ammonia to generate hydrazine^54^; it is possible that *Ca.* Brocadia can bypass NO entirely and deliver hydroxylamine directly to Hzs.

In contrast, *Ca.* Kuenenia*, Ca.* Scalindua, and *Ca.* Jettenia all include a canonical nitrite reductase in their genomes. Indeed, Kobayashi and coworkers^19^ observed that the offset in ^15^ε(NO_2_^–^–N_2_) between measured values for *Ca.* Kuenenia and *Ca.* Scalindua, which have the iron-bearing nitrite reductase NirS, and *Ca.* Jettenia, which has the copper-bearing nitrite reductase NirK, corresponds to that observed for NirK and NirS in bacterial denitrifiers^55^. This interpretation is complicated by the observation that the genes for these canonical nitrite reductases are often not expressed^49,56^ or translated^57^ under environmental conditions. Nevertheless, the differences in N isotopic discrimination of nitrite among anammox clades appear to correspond to fundamental differences in the conversion of nitrite, but the molecular mechanisms of these steps remain poorly understood.

### Inverse isotope effect imparted in ^15^ε(NO_2_^–^–NO_3_^–^) by nitrite oxidation

A pronounced inverse isotope effect, in which nitrate becomes enriched in ^15^ N relative to nitrite from which it is produced, was observed in all experimental settings. Such an inverse isotope effect appears to be a signature feature of microbial nitrite oxidation to nitrate, both under oxic and anoxic (*i.e.*, anammox) conditions^58,59^ In culture-based experiments with nitrite oxidizing bacteria (NOB), ^15^ε(NO_2_^–^–NO_3_^–^) has been found to vary between − 7.8‰ and − 23.6‰^59,60^, while anammox bacteria have been shown to express ^15^ε(NO_2_^–^–NO_3_^–^) values in pure or highly-enriched cultures between − 30 and − 45‰^10,19^, with values as low as − 78‰ in a wastewater incubation experiment^20^. In our experiments, we found N isotope effects that cover nearly this whole range (Fig. 4). In both anammox and the NOB, nitrite oxidation is thought to be performed by the enzyme nitrate:nitrite oxidoreductase (Nxr)^25,43,59^, which is also closely related to bacterial membrane-bound and periplasmic nitrate reductases^61,62^. The structural details of the Nxr enzyme family are not yet well explored, especially in light of its diverse metabolic roles^63^, and so it remains unclear what metabolic or microbial processes are responsible for the observed and reported variation in ^15^ε(NO_2_^–^–NO_3_^–^). At least for anammox bacteria, the inverse kinetic N isotope effect associated with the enzymatic oxidation of nitrite to nitrate may be superposed in part by a relatively large equilibrium N isotope effect between nitrite and nitrate^10^, perhaps promoted by the reversibility of the enzymatic nitrite oxidation reaction^64^. It is notable that the most negative end of the observed range for ^15^ε(NO_2_^–^–NO_3_^–^) in this study approaches the theoretical limit for the isotope effect set by the N isotope equilibrium between nitrite and nitrate, which at 20 °C is – 54.6‰^59^, and which the NOB have not been observed to approach. This suggests that under the metabolic conditions of anammox, the Nxr enzyme is more likely to catalyze reversible reactions, and so the corresponding N isotope effect is closer to the equilibrium limit, than in aerobic nitrite oxidation. However, the great range observed in ^15^ε(NO_2_^–^–NO_3_^–^) for anammox makes it difficult to predict a priori how much fractionation anammox will impart on a nitrate pool.

On the other hand, observations in this study and elsewhere^10,19^ (Fig. 4) of values of ^15^ε(NO_2_^–^–NO_3_^–^) falling near − 30‰ for nitrate generated by anammox match a prediction from water column measurements of N isotope ratios in nitrate and nitrite in the Peru oxygen deficient zone (ODZ)^65^. The large and variable magnitude of this inverse isotope effect means that even though only ~ 25% of the nitrite oxidized by anammox is converted to nitrate, it can have an outsize effect on nitrate and nitrite pools that can be mistaken for either nitrite oxidation by NOB or nitrite generation by denitrification.

### Implications for N isotope measurements in natural and engineered environments

Taken together, the results measured in this study suggest both potential and pitfalls for the application of N isotope measurements to disentangle the systematics of microbial N cycling processes. By its interaction with the nitrate, nitrite, ammonium, and N_2_ pools, anammox already complicates analysis of the N cycle in any setting where it acts; not only does it impact the stable isotope pools of these molecules, but also its effects on each of these pools can vary greatly depending on physiological or metabolic variables or the identity of the dominant anammox bacteria species. In the context of a wastewater treatment process, measurements of δ^15^N alone may not be able to directly diagnose what processes are occurring, but when coupled to rate and stoichiometry measurements, may provide insights into the efficiency or limitations of those processes.

The removal of ^15^ N-depleted N by anammox may partly explain the heavy N isotope values (> 15‰) for nitrate in ODZs that have been formerly attributed to denitrification alone. It remains uncertain, however, what the exact expression of the range of N isotope effects reported here is under natural and variable substrate concentrations. First, as shown here for ammonium, concentration levels will have an effect on the relative kinetics of uptake and oxidation, and in turn on the cell-specific N isotope effect. Second, ammonium concentrations are generally at or below detection in the interior of OMZs, indicating that ammonium supplied by degradation of organic matter may be quantitatively oxidized to N_2_ by anammox. Under these conditions, the N isotope effect associated with the conversion of ammonium to N_2_ will be suppressed. Previously published estimates of the overall N isotope effect of dissolved inorganic N (DIN) elimination to N_2_ in ODZs based on comparing nitrate δ^15^N values to observed water-column nitrate deficits has inherently included any potential non-fractionating loss of ammonium, and has thus implicitly represented a community N loss isotope effect that depends on the balance between anammox and canonical denitrification. The overall expression on DIN lost by the combined processes of denitrification and anammox in sediments, however, may be completely different. In contrast to nitrate and nitrite^66^, ammonium is usually not limiting in sediments and its fractional loss to overlying waters allows the N isotope effect of ammonium oxidation to N_2_ by benthic anammox to be expressed.

In both natural and engineered systems where anammox is known to be occurring, measurements of ^15^ε(NH_4_^+^) may be able to diagnose substrate limitations or other physiological limitations. And in the case where ammonium is observed to be consumed by an unknown pathway, it can be expected that isotope effects will fall into a similar range for both aerobic and anaerobic ammonium oxidation; on the other hand, ^15^ε(NH_4_^+^) is of little use for distinguishing ammonium consumption by AOB and anammox. We have also found that despite the great possibility for variation in ^15^ε(NO_2_^–^–N_2_) amongst different anammox species, values of ^15^ε(NO_2_^–^–N_2_) remain relatively stable in a given system that has a stable microbial community, and so this parameter has some potential to be used in monitoring such microbial community stability. Further work is needed to explore how metabolic variation amongst anammox species is related to variations in this parameter, but it appears that subtle changes in the mix of anammox species present, which have no observed effect on anammox rates or stoichiometry can lead to major changes in ^15^ε(NO_2_^–^–N_2_). Finally, this study expands the range of ^15^ε(NO_2_^–^–NO_3_^–^) for anammox bacteria. On one hand, this result lends further support to the observation that through a strong manifestation of the inverse isotope effect associated with nitrite oxidation, anammox can produce nitrate strongly enriched in ^15^ N, thereby complicating N mass balances based on tracking the nitrate pool. But we also find that anammox can have ^15^ε(NO_2_^–^–NO_3_^–^) values much closer to 0‰, falling in the same range as for NOB bacteria, so the contribution of anammox to the δ^15^N composition of the nitrate pool can in fact vary greatly. Finally, we find that there are no systematic relationships among ^15^ε(NH_4_^+^), ^15^ε(NO_2_^–^–N_2_), ^15^ε(NO_2_^–^–NO_3_^–^), which is consistent with the conclusion that each of these parameters is controlled at a distinct point in the anammox metabolism.

## Supplementary Information

Supplementary Information 1.

Supplementary Information 2.
