# Supplementary material for: Nitrogen isotope effects can be used to diagnose N transformations in wastewater anammox systems
Source: Sci Rep. 2021 Apr 12;11:7850. doi: 10.1038/s41598-021-87184-0 (PMC8041819; doi:10.1038/s41598-021-87184-0)
Supplement: Supplementary file 2 — Supplementary Information 2. [file 41598_2021_87184_MOESM2_ESM.docx]

SUPPLEMENTARY INFORMATION

Nitrogen isotope effects can be used to diagnose N transformations in wastewater anammox systems

Paul M. Magyar, Damian Hausherr, Robert Niederdorfer, Nicolas Stöcklin, Jing Wei, Joachim Mohn, Helmut Bürgmann, Adriano Joss, and Moritz F. Lehmann

Figure S1. Microbial community composition, at the Class level. Anammox bacteria fall into the Class Brocadiae. Data from Niederdorfer and coworkers.^23^


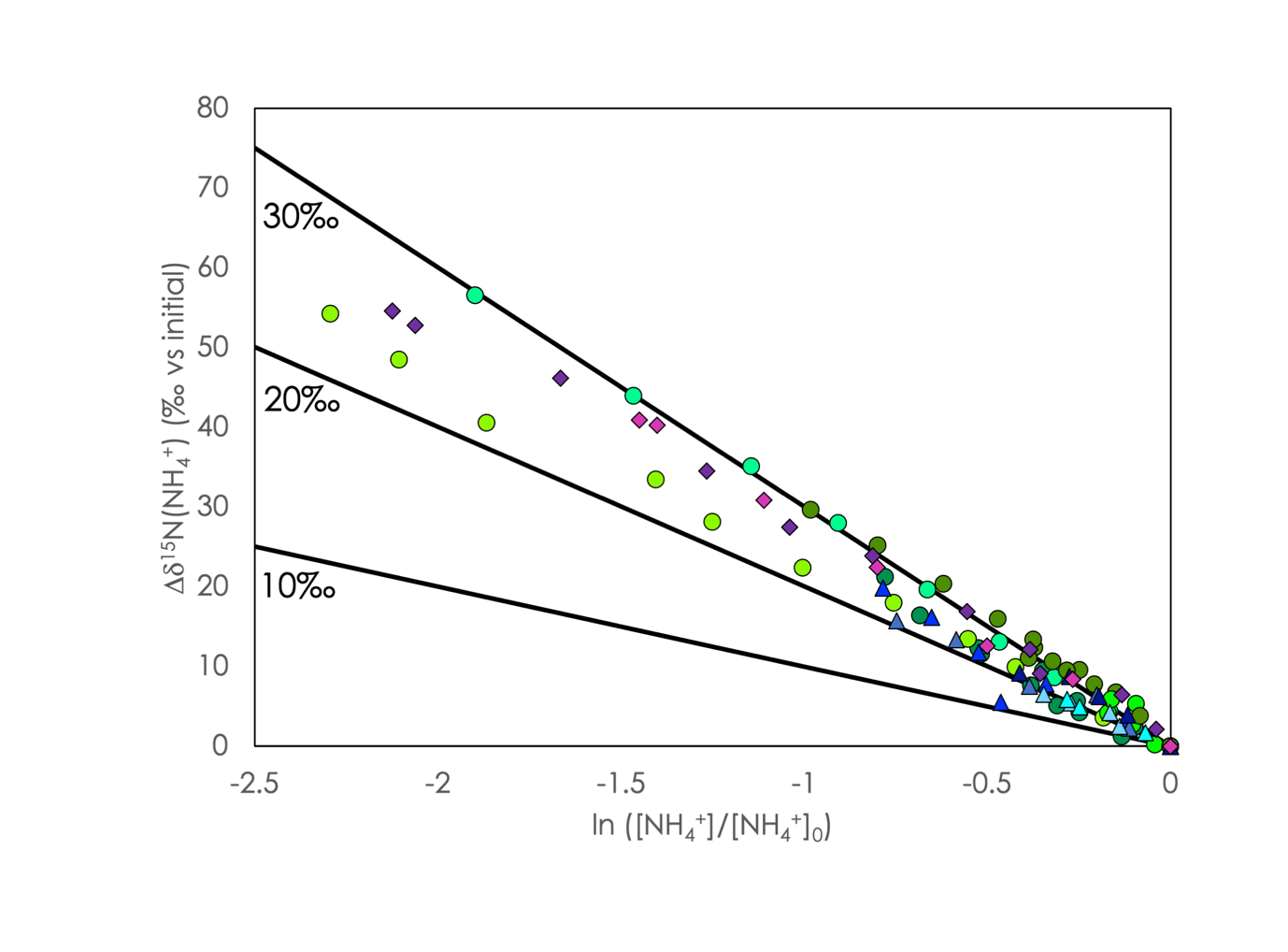


Figure S2. Rayleigh plot for the distillation of ammonium in anammox incubation experiments. Mainstream experiments are represented by triangles and shades of blue, enrichment experiments by circles and shades of green, and sidestream experiments by diamonds and shades of purple. For reference, the slopes associated with ^15^ε(NH_4_^+^) values of 10‰, 20‰, and 30‰ are also shown. Regression lines described in the main text are calculated for each anammox culture.


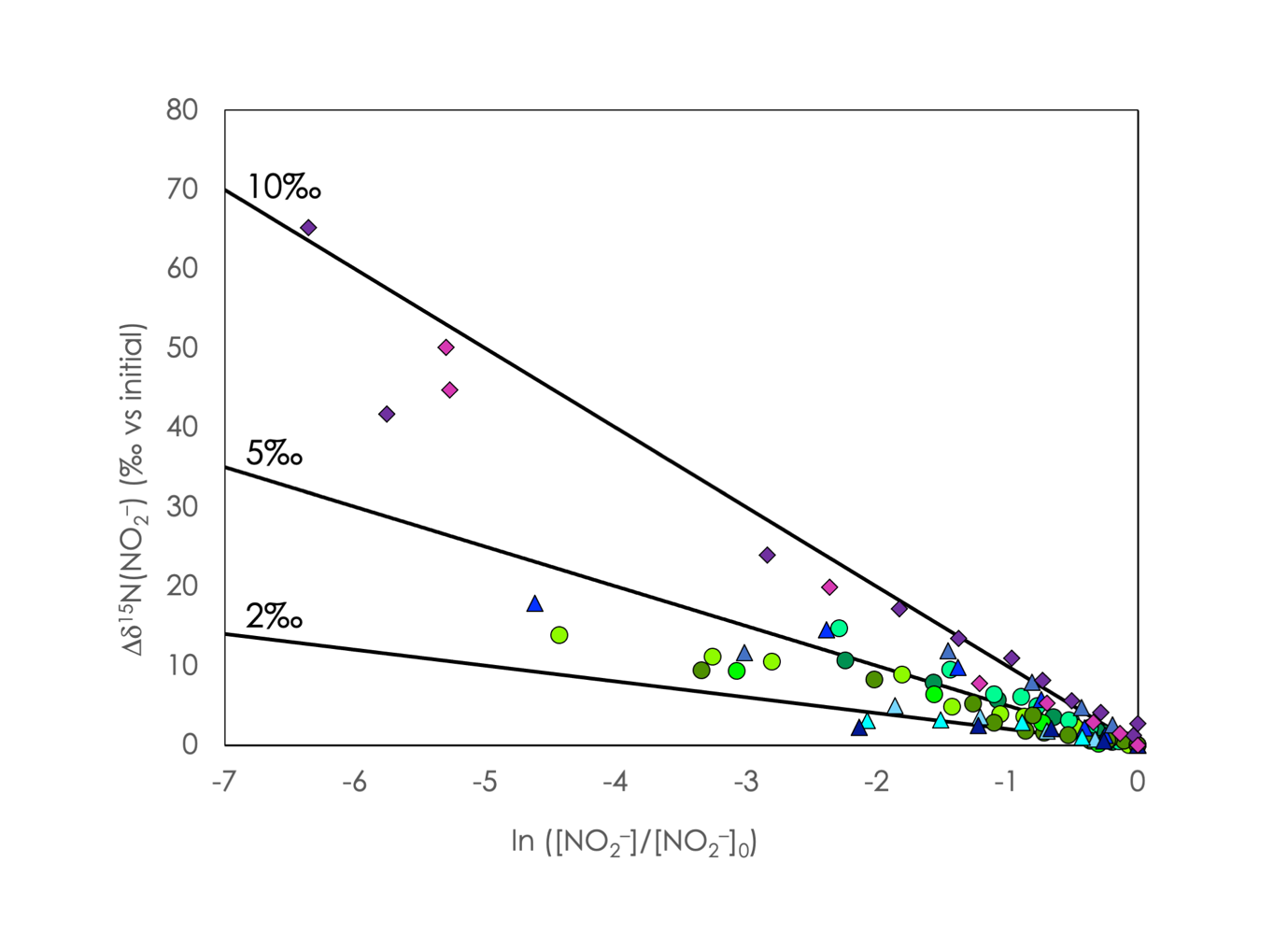


Figure S3. Rayleigh plot for the distillation of nitrite in anammox incubations. This process convolves the reduction of nitrite to N_2_ and the oxidation of nitrite to nitrate into a single number. Mainstream experiments are denoted by triangles, enrichment experiments by circles, and sidestream experiments by diamonds. For reference, the slopes associated with ^15^ε(NO_2_^-^) values of 2‰, 5‰, and 10‰ are also shown. Regression lines described in the main text are calculated for each anammox culture.


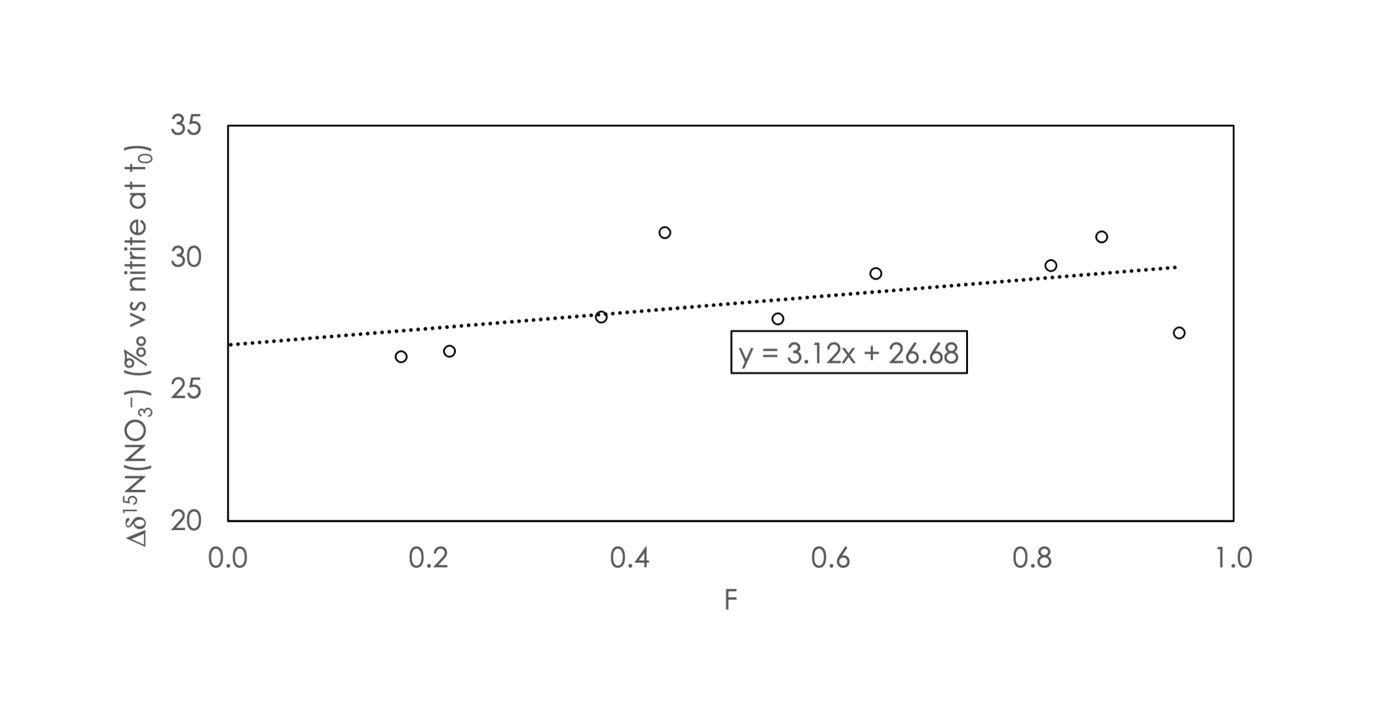


Figure S4. The δ^15^N of nitrate, for a sample experiment (enrichment, 28 January 2019), corrected for the amount and isotopic composition of nitrate that was in the reactor before the experiment started, and normalized to δ^15^N(NO_2_^-^)_0_, the initial δ^15^N of nitrite in the reactor. δ^15^N(NO_3_^-^) is plotted against the parameter F, which tracks the accumulation of nitrate, as described in the main text. The y-intercept corresponds to the isotope effect for the formation of nitrate from nitrite, ^15^ε(NO_2_^–^–NO_3_^–^), in this example 26.7‰.

|  | Ammonium consumption rate, (mg-N L^-1^ min^-1^) | Nitrite consumption rate (mg-N L^-1^ min^-1^) | ∆NO_2_^–^/∆NH_4_^+^ | ∆NO_3_^–^/∆NO_2_^–^ | Starting NH_4_^+^ concentrations (mg-N L^-1^) | Starting NO_2_^–^ concentrations (mg-N L^-1^) |
| --- | --- | --- | --- | --- | --- | --- |
| Mainstream | 0.16 ± 0.02 | 0.21 ± 0.16 | 1.30 ± 0.10 | -0.22 ± 0.10 | 25.0 to 56.0 | 17.5 to 43.6 |
| Enrichment | 0.79 ± 0.16 | 1.16 ± 0.79 | 1.42 ± 0.16 | -0.22 ± 0.05 | 63.8 to 195 | 45.9 to 113 |
| Sidestream | 0.49 ± 0.07 | 0.68 ± 0.49 | 1.37 ± 0.09 | -0.162 ± 0.002 | 61.4 to 87.1 | 72.8 to 85.0 |

Table 1. Summary of reaction rates and stoichiometries for all experimental systems. Values are the average of all experiments reported for each system; error estimates are ±1 standard deviation.

| Species | Proportional Abundance (%) |
| --- | --- |
| *Candidatus* Brocadia caroliniensis | 40.6% |
| *Candidatus* Brocadia sinica | 25.6% |
| *Candidatus* Brocadia sapporoensis | 6.1% |
| *Candidatus* Brocadia sp. UTAMX1 | 6.2% |
| *Candidatus* Brocadia fulgida | 5.4% |
| *Candidatus* Kuenenia stuttgartiensis | 5.2% |
| *Candidatus* Jettenia caeni | 4.7% |

Table S1. Identities and proportional abundances of the 8 anammox species observed in metagenomic analyses of the mainstream anammox system.^22^
